# Most patients with tibial plateau fractures regain function for daily activities but do not return to their pre-injury level of sport: comparison of multicenter cohort of 1101 patients and age-related peers

**DOI:** 10.1007/s00068-025-02958-9

**Published:** 2025-09-03

**Authors:** Thijs P. Vaartjes, Fabian J. van der Sluis, Eelke Bosma, Joost G. ten Brinke, Reinier de Groot, Job N. Doornberg, Harm Hoekstra, Frank F. A. IJpma, Nick Assink

**Affiliations:** 1 Department of Trauma Surgery, University of Groningen, University Medical Center Groningen, HPC BA13, Hanzeplein 1, Groningen, 9713 GZ The Netherlands; 2 Department of Trauma Surgery, Isala hospital, Zwolle, The Netherlands; 3 Department of Trauma Surgery, Martini hospital, Groningen, The Netherlands; 4 Department of Trauma Surgery, Gelre hospitals, Apeldoorn, Zutphen, The Netherlands; 5 Department of Trauma Surgery, Medical Spectrum Twente, Enschede, The Netherlands; 6 Department of Orthopaedic Surgery, University of Groningen, University Medical Center Groningen, Groningen, The Netherlands; 7 Department of Trauma Surgery, University Hospitals Leuven, Leuven, Belgium; 8 3D lab, University of Groningen, University Medical Center Groningen, Groningen, The Netherlands

**Keywords:** Tibial plateau fractures, KOOS, Patient-reported outcome, Outcome age-related peers, Sport

## Abstract

**Purpose:**

The aim was to evaluate patient-reported outcomes - symptoms, pain, activities of daily living (ADL), sports, and quality of life (QoL) - in patients with tibial plateau fractures. Outcomes were compared with those of age-matched peers from the general population to support patient counseling.

**Methods:**

A multicenter cross-sectional study was performed including 1101 patients with tibial plateau fractures between 2003 and 2019. At mean follow-up of 6 ± 4 years, patients completed the Knee injury and Osteoarthritis Outcome Score (KOOS) questionnaire. Patients were grouped by age, treatment, and Schatzker classification. Summery Independent-Samples t-tests were used to compare patients KOOS values with age-related peers reference values. Descriptive statistics were used to report the proportion of patients who recovered within the minimal clinically important difference (MCID) range.

**Results:**

Nonoperatively treated patients scored 5% lower than age-related peers for symptoms, pain and ADL. These differences did not exceed the MCID, and 68% of patients regained the level of age-related peers. Patients < 60 years scored 22% lower for sports and QoL, with 45% and 53% of patients recovering to peer levels. Operatively treated patients scored 14% lower compared to age-related peers for symptoms, pain and ADL, exceeding the MCID, and 53% of patients regained the level of peers. For sports and QoL, scores were 36% lower, with only 30%, and 39% of patients returning to peer levels.

**Conclusion:**

While most patients regain satisfactory function in daily activities after tibial plateau fractures, many—especially those treated operatively—do not return to pre-injury levels of sports and QoL. Our findings provide a guideline for managing patients’ expectations among different age-groups.

**Level of evidence:**

Prognostic Level III.

**Supplementary Information:**

The online version contains supplementary material available at 10.1007/s00068-025-02958-9.

## Introduction

Tibial plateau fractures account for 1–2% of all fractures and are intra-articular fractures that are challenging to treat [1, 2]. Since the tibial plateau is among the most loadbearing areas in the body, these fractures can have a major impact on patient’s life. At short term these injuries are mainly disabling due to immobilization or postoperative recovery. In the long term, these patients are at higher risks of post-traumatic osteoarthritis, chronic pain, knee stiffness or instability, which may ultimately result in the conversion to a total knee arthroplasty (TKA) [3–7]. Studies reporting on patient-reported functional outcome after tibial plateau fractures have shown contradictory results. While some studies report satisfactory outcomes during follow-up, others highlight poorer outcomes after both operative and nonoperative treatment [8–13].

Although functional outcomes after tibial plateau fractures have been studied extensively, it remains unclear how well patients recover over time and whether their outcomes match those of age-matched peers in the general population. Recently, van den Berg et al. compared functional outcomes of patients with tibial plateau fractures with reference values of the general population [10]. They reported that outcomes after both operative and nonoperative treatment were significantly lower compared to those of the reference population. Similar results were observed in the study by Timmer et al. who reported fair functional outcomes in surgically treated patients, although these were lower compared to the general Dutch population [8]. However, these studies compared patient-reported functional outcomes of the whole study population without accounting for the patient’s age and fracture types. Literature on population-based reference values for functional outcome scores indicate that functional outcomes vary across different age groups within the general population [14, 15]. This could be due to differences in level of activity, ambulatory status, preexisting comorbidities, and coping mechanisms between younger and older individuals. To adequately counsel patients during treatment it is important to understand to what extent they will recover during follow-up and how their outcomes compare to those of age-related peers in the general population.

In this study, we aim to assess the differences in functional outcome after tibial plateau fractures compared with those of the age-related general population. Assuming that healthy peers from the general population represents the pre-injury status, every patient wants to know to what extent they will recovery relative to this healthy baseline. Also, younger patients often have different levels of physical functioning and expectations compared to older patients, highlighting the importance of assessing outcomes across different age groups. The goal of this study is to provide a guideline for patient counselling by describing functional outcomes following tibial plateau fractures in relation to healthy peers, stratified by age group and fracture type. This information could be helpful to manage patient’s expectations more accurately. Therefore, we posed the following research question: How do patient-reported outcomes regarding knee symptoms, pain, daily activities, sports and quality of life differ between patients who sustained a tibial plateau fracture and age-related peers from the general population?

## Methods

### Study design

A multicenter cross-sectional study was performed on all patients treated for a tibial plateau fracture in the orthopaedic or trauma surgery department of six trauma centers in two countries (University Medical Center Groningen, Isala hospitals, Gelre hospitals, Martini hospital, Medical Spectrum Twente and KU Leuven University Hospitals) between January 2003 and December 2019. Patients were eligible for inclusion if they had a preoperative CT scan available, had knowledge of the Dutch language, were alive at the time of follow-up and had at least one year follow-up. Patients < 18 years of age at time of the accident, with compromising injuries (e.g. severe concomitant injuries on the same leg or paralysis), complicated fractures requiring amputation or had an unknown address at time of follow-up were excluded. Baseline characteristics were retrieved from the patients’ electronic records. Knee radiographs and CT images taken within 2 weeks after the patient’s injury were reassessed and classified through consensus by two observers (TPV, FFAIJ) with experience in tibial plateau fracture management according to the Schatzker classification systems [16]. All eligible patients were approached by posted mail and asked for written informed consent and to complete the validated patient-reported outcome measures.

The study procedure was approved by the institutional review board of all participating centers (number: 201800411) and was performed in accordance with the relevant guidelines and regulations. This study is reported in accordance with the Strengthening the Reporting of Observational Studies in Epidemiology (STROBE) guideline [17].

### Patient-reported outcomes

All responding patients completed the Knee Injury and Osteoarthritis Outcome Score (KOOS) questionnaire [18]. The validated and standardized KOOS questionnaire consists of five subscales: symptoms, pain, activities of daily living (ADL), function in sports and recreation (sport), and knee-related quality of life (QoL). The total score was calculated for each subscale by adding the scores of individual items (i.e. questions) and transforming them to a range from 0 to 100, with higher scores indicating better function. In addition, patients were asked whether they underwent conversion to TKA. Patients with conversion to TKA were assigned an average KOOS score based on a previously reported cohort of patients’ KOOS scores measured just before conversion to TKA [19]. The reported KOOS scores of each subscale were 52 for symptoms, 45 for pain, 55 for ADL, 16 for sport and 27 for QoL. The minimal clinically important difference (MCID) of each subscale from the KOOS questionnaire is 9 for symptoms, 12 for pain, 10 for ADL, 9 for sports, and 14 for QoL [20]. Functional outcomes of age-related peers were retrieved from a previous cohort that described National record-based reference values for the KOOS questionnaire of the general population in Denmark [15].

### Statistical analysis

IBM SPSS software, version 23.0 for Windows (IBM Corporation, Chicago, IL, USA) was used for statistical analysis and figures were created with Microsoft Excel 365 (Version 2025, Microsoft Corporation, Redmond, WA, USA). Continuous variables are presented as the mean and standard deviation (SD) for normally distributed data, and median and inter-quartile range (IQR) for nonnormally distributed data. Descriptive statistics were used to describe the study population. The study population was divided into six groups based on age (18–29, 30–39, 40–49, 50–59, 60–69 and 70+). Analysis of variance (ANOVA) was applied to compare continuous variables, while the chi-square test was used for categorical variables to assess differences in patient and fracture characteristics across age groups. The study population was further divided based on treatment (operative or nonoperative) and a Summery Independent-Samples t-test was used to assess differences in patient-reported functional outcome between patients and age-related peers. Descriptive statistics were used to describe the number of patients who had recovered after operative or nonoperative treatment, defined as having KOOS-score within the MCID range of age-related peers [15]. For sub-analysis the study population was divided based on age and fracture classification according to Schatzker [16]. A p-value of less than 0.05 was considered statistically significant.

## Results

Between 2003 and 2019, a total of 2288 patients were treated for a tibial plateau fracture within six hospitals. Of these patients, 144 were < 18 years, 89 had compromising injuries, 4 had an amputation and 60 had an unknown address, leaving 1991 patients eligible for follow-up analysis. A total of 55% (1101 of 1991) of patients responded to the questionnaire and were included in the study. The mean age at the time of injury was 53 ± 15 years, 68% (751 of 1101) of patients were female, and the mean follow-up was 6 ± 4 years. A total of 756 out of 1101 (69%) patients were treated operatively by using plate and/or screw osteosynthesis. Eventually, 123 (11%) patients underwent conversion to TKA during follow-up (Table 1).

**Table 1:** Demographics

Age group	18–29*	30–39*	40–49*	50–59*	60–69*	70+*	*P*-value
Number of patients	105	102	158	329	279	128	
Age (years)	24 ± 3	35 ± 3	45 ± 3	55 ± 3	64 ± 3	75 ± 5	0.000
Sex (Female)	56 (53%)	48 (47%)	85 (54%)	232 (71%)	219 (79%)	111 (87%)	< 0.001
Body Mass Index	25 ± 6	26 ± 5	28 ± 5	27 ± 4	26 ± 4	26 ± 4	< 0.001
Smoking	29 (28%)	30 (30%)	43 (27%)	72 (22%)	40 (15%)	6 (5%)	< 0.001
Diabetic	3 (3%)	4 (4%)	9 (6%)	27 (8%)	31 (11%)	12 (10%)	0.036
Treatment							0.084
Operative	70 (67%)	76 (75%)	113 (72%)	236 (72%)	185 (66%)	76 (59%)	
Nonoperative	35 (33%)	26 (26%)	45 (29%)	93 (28%)	94 (34%)	52 (41%)	
Trauma mechanism							< 0.001
Low energy trauma	80 (80%)	85 (85%)	124 (82%)	282 (88%)	252 (93%)	120 (95%)	
High energy trauma	20 (20%)	15 (15%)	27 (18%)	37 (12%)	18 (7%)	6 (5%)	
Fracture classification							0.001
Schatzker I	14 (13%)	10 (10%)	13 (8%)	21 (6%)	11 (4%)	8 (6%)	
Schatzker II	36 (34%)	26 (26%)	58 (37%)	114 (35%)	105 (38%)	45 (35%)	
Schatzker III	24 (23%)	28 (28%)	26 (17%)	72 (22%)	67 (24%)	33 (26%)	
Schatzker IV	12 (11%)	24 (24%)	29 (18%)	37 (11%)	36 (13%)	20 (16%)	
Schatzker V	13 (12%)	4 (4%)	10 (6%)	28 (9%)	12 (4%)	6 (5%)	
Schatzker VI	6 (6%)	10 (10%)	22 (14%)	57 (17%)	48 (17%)	16 (13%)	
Gap (mm)	5.0 ± 5.7	5.4 ± 6.0	5.1 ± 4.8	5.9 ± 6.1	5.1 ± 5.3	5.4 ± 5.2	0.552
Step-off (mm)	5.6 ± 6.4	6.2 ± 6.5	5.8 ± 5.2	6.8 ± 6.7	5.7 ± 5.5	6.2 ± 6.4	0.257
Follow-up (years)	7 ± 4	8 ± 5	7 ± 4	6 ± 4	6 ± 4	5 ± 3	< 0.001
Conversion to TKA	0 (0%)	4 (4%)	9 (6%)	40 (12%)	44 (16%)	26 (20%)	< 0.001

* Numbers are presented as mean ± standard deviation or as number and percentage

Non-response analysis demonstrated that non-responders were slightly younger (51 ± 17 vs. 53 ± 15, *P* = < 0.001), less often female (58% vs. 68%, *P* = < 0.001), and less often treated surgically (57% vs. 69%, *P* = < 0.001) in comparison with responders. There was no difference in fracture classification between responders and non-responders (Schatzker I 3% versus 5%; Schatzker II 39% versus 36%; Schatzker III 11% versus 7%; Schatzker IV 12% versus 16%; Schatzker V 10% versus 9%; Schatzker VI 26% versus 28% *p* = 0.618).

### Level of recovery as compared to age-related peers

**Fig. 1:**
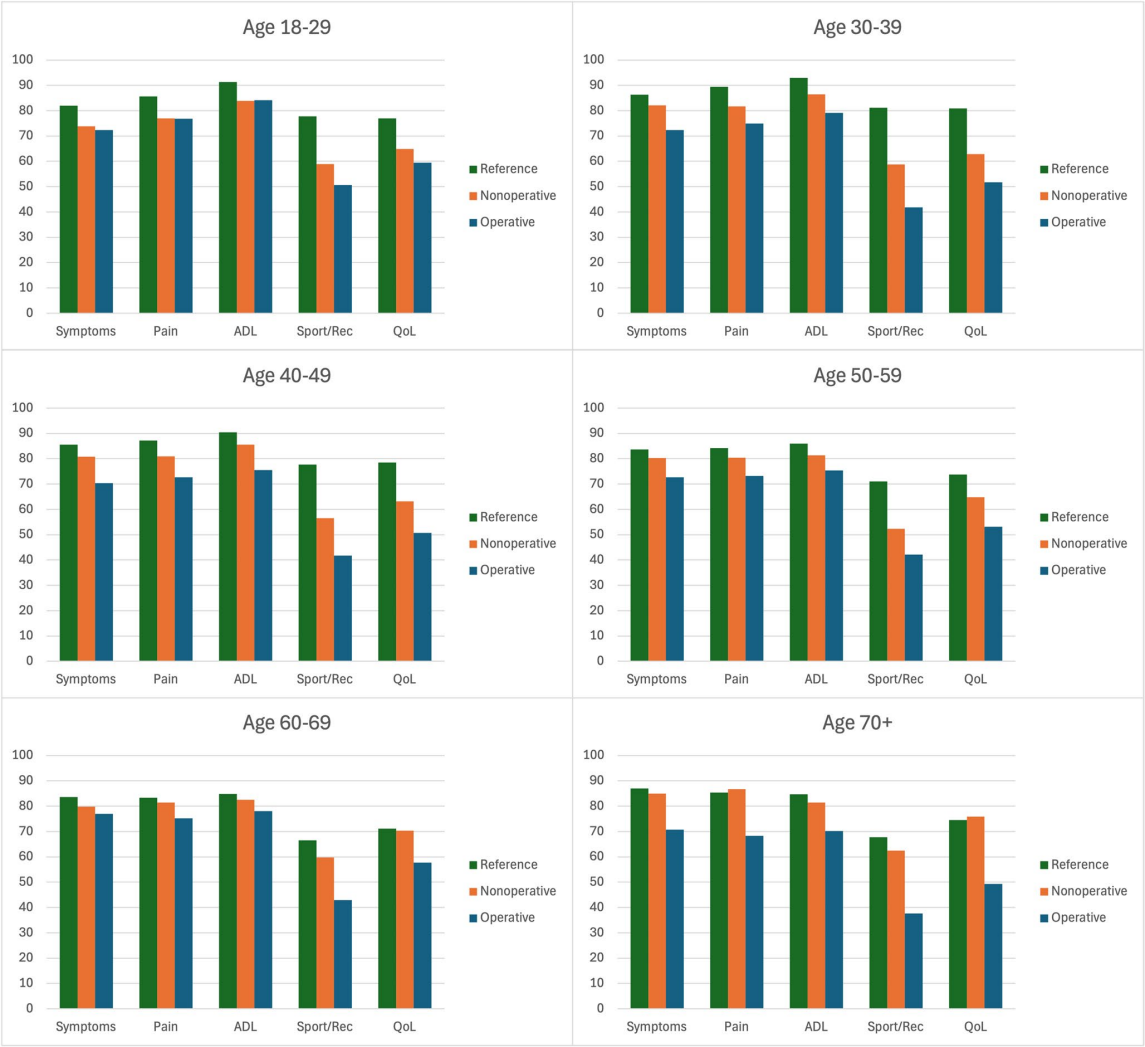
The average KOOS-score for each subscale of the different age and treatment groups compared with age-related peers

The average KOOS-scores were lower in most subscales for both operatively and nonoperatively treated patients with tibial plateau fractures as compared to their age-related peers from the general population (Fig. 1).

Nonoperatively treated patients scored 5% lower for the subscales symptoms, pain and ADL, but these differences did not exceed the minimal clinical important difference (MCID) (Table 2). For these same subscales, 60–81% of patient recovered well and reached the level of age-related peers within the MCID range (Table 2). For the subscales sports and recreation as well as quality of life, patients younger than 60 years scored 22% lower compared to peers from the general population, with differences exceeding the MCID in these younger age groups. For sports and recreation, only 41–49% of the younger patients (< 60 years) and for quality of life only 49–58% of younger patients reached the level of age-related peers within the MCID range (Table 2).

**Table 2:** The average KOOS-score of each age group after nonoperative treatment compared with age-related peers

Nonoperative	Outcome score after Tibial plateau fracture		Outcome score general population			Difference on a scale of 0-100	
Age group	Number of patients	KOOS Symptoms*	Number of peers	KOOS Symptoms*	*P*-value	MCID 9**	Number of recovered patients ***
18–29	35	73.8 ± 21.7	153	82.0 ± 18.1	0.021	−8,2	21 (60%)
30–39	26	82.1 ± 16.8	218	86.3 ± 14.7	0.176	−4,2	17 (65%)
40–49	45	80.8 ± 15.2	351	85.5 ± 16.3	0.067	−4,7	28 (62%)
50–59	93	80.2 ± 19.2	473	83.7 ± 17.3	0.081	−3,5	62 (67%)
60–69	93	79.8 ± 21.1	640	83.6 ± 17.3	0.100	−3,8	62 (66%)
70+	52	85.0 ± 19.7	1007	87.0 ± 16.2	0.391	−2,0	41 (79%)
Age group	Number of patients	KOOS Pain*	Number of peers	KOOS Pain*	*P*-value	MCID 12 **	Number of recovered patients ***
18–29	35	76.9 ± 20.3	153	85.6 ± 18.2	0.013	−8,7	22 (63%)
30–39	26	81.7 ± 18.9	218	89.4 ± 15.3	0.019	−7,7	18 (69%)
40–49	45	80.9 ± 19.3	351	87.2 ± 17.1	0.022	−6,3	27 (60%)
50–59	93	80.4 ± 22.5	473	84.2 ± 19.0	0.088	−3,8	64 (69%)
60–69	92	81.5 ± 21.8	640	83.3 ± 19.3	0.411	−1,8	65 (71%)
70+	52	86.8 ± 19.2	1004	85.4 ± 18.0	0.586	+ 1,4	42 (81%)
Age group	Number of patients	KOOS ADL*	Number of peers	KOOS ADL*	*P*-value	MCID 10 **	Number of recovered patients ***
18–29	35	83.9 ± 19.5	152	91.3 ± 14.1	0.040	−7,4	24 (69%)
30–39	26	86.4 ± 18.3	217	93.0 ± 12.5	0.084	−6,6	18 (69%)
40–49	45	85.6 ± 16.7	350	90.4 ± 15.3	0.051	−4,8	30 (67%)
50–59	93	81.3 ± 20.9	473	85.9 ± 18.4	0.050	−4,6	62 (67%)
60–69	93	82.6 ± 20.0	639	84.8 ± 18.1	0.280	−2,2	63 (68%)
70+	52	81.5 ± 26.6	998	84.7 ± 18.1	0.395	−3,3	39 (75%)
Age group	Number of patients	KOOS Sport*	Number of peers	KOOS Sport*	*P*-value	MCID 9 **	Number of recovered patients ***
18–29	35	58.9 ± 33.4	152	77.8 ± 27.1	0.003	**−18**,**9**	17 (49%)
30–39	25	58.8 ± 38.4	217	81.2 ± 25.1	0.008	**−22**,**4**	12 (48%)
40–49	45	56.5 ± 34.1	348	77.7 ± 27.3	< 0.001	**−21**,**2**	19 (42%)
50–59	91	52.3 ± 35.7	469	71.1 ± 29.9	< 0.001	**−18**,**8**	37 (41%)
60–69	88	59.8 ± 33.0	631	66.5 ± 31.4	0.063	−6,7	50 (57%)
70+	43	62.5 ± 36.8	982	67.8 ± 31.2	0.280	−5,3	27 (63%)
Age group	Number of patients	KOOS Quality of life*	Number of peers	KOOS Quality of life*	*P*-value	MCID 14 **	Number of recovered patients ***
18–29	35	64.9 ± 24.6	152	76.9 ± 25.0	0.011	−12,0	18 (51%)
30–39	25	62.8 ± 24.4	217	80.8 ± 23.2	< 0.001	**−18**,**0**	13 (52%)
40–49	45	63.2 ± 27.3	351	78.5 ± 23.0	< 0.001	**−15**,**3**	22 (49%)
50–59	93	64.8 ± 27.6	473	73.8 ± 25.9	0.003	−9,0	54 (58%)
60–69	93	70.3 ± 27.3	640	71.2 ± 26.4	0.760	−0,9	61 (66%)
70+	51	75.9 ± 28.6	996	74.6 ± 25.6	0.725	+ 1,3	38 (75%)

* The values are given as mean and standard deviation.

** The cursive numbers indicate KOOS-scores exceeding the minimal clinically important difference (MCID)

*** The number and percentage of patients that recovered within the MCID range of the outcome scores from age-related peers.

**Fig. 2:**
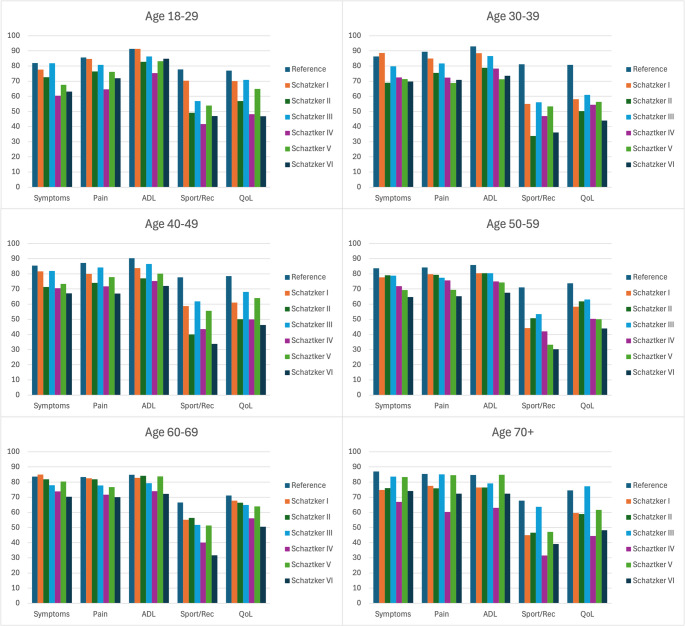
The average KOOS-score for each subscale of the different age groups stratified by Schatzker classification compared with age-related peers

Operative treated patients scored 14% lower than peers on the symptoms, pain and ADL subscales. In most age groups, the scores slightly exceeded the minimal clinical important difference (MCID) (Table 3). For these subscales, 43–67% of patients recovered well and reached the level of age-related peers within the MCID range (Table 3). For the sports and recreation as well as quality of life, scores were substantially lower, by approximately 36% compared to peers, and these differences exceeded the MCID across all age groups. For sports and recreation, and quality of life, 26–35% and 28–47% of patients reached the level of age-related peers within the MCID range, respectively (Table 3). After tibial plateau fractures, sports and recreation as well as quality of life were the most affected, which is particularly pronounced in younger age groups (Tables 2 and 3). Having established a general understanding of how patients in specific age categories perform compared to their age-related peers, it is important to recognize that fracture complexity varies within these age and treatment groups. Table 1 in the supplementary data presents patient functioning stratified by Schatzker classification, compared to age-related peers. Overall, higher Schatzker classifications, which reflect more severe fractures, are associated with lower expected functional recovery (Fig. 2 appendix).

**Table 3:** The average KOOS-score of each age group after operative treatment compared with age-related peers

Operative	Outcome score after Tibial plateau fracture		Outcome score general population			Difference on a scale of 0-100	
Age group	Number of patients	KOOS Symptoms*	Number of peers	KOOS Symptoms*	P-value	MCID 9**	Number of recovered patients ***
18–29	70	72.3 ± 20.4	153	82.0 ± 18.1	< 0.001	**−9**,**7**	37 (53%)
30–39	76	72.4 ± 21.9	218	86.3 ± 14.7	< 0.001	**−13**,**9**	34 (45%)
40–49	113	70.3 ± 22.1	351	85.5 ± 16.3	< 0.001	**−15**,**2**	52 (46%)
50–59	236	72.7 ± 21.8	473	83.7 ± 17.3	< 0.001	**−11**,**0**	119 (50%)
60–69	184	77.0 ± 19.3	640	83.6 ± 17.3	< 0.001	−6,6	109 (59%)
70+	75	70.6 ± 22.7	1007	87.0 ± 16.2	< 0.001	**−16**,**3**	32 (43%)
Age group	Number of patients	KOOS Pain*	Number of peers	KOOS Pain*	P-value	MCID 12 **	Number of recovered patients ***
18–29	70	76.8 ± 20.0	153	85.6 ± 18.2	0.001	−8,8	42 (60%)
30–39	76	74.9 ± 23.2	218	89.4 ± 15.3	< 0.001	**−14**,**5**	43 (57%)
40–49	113	72.7 ± 22.6	351	87.2 ± 17.1	< 0.001	**−14**,**5**	56 (50%)
50–59	236	73.2 ± 23.8	473	84.2 ± 19.0	< 0.001	−11,0	133 (56%)
60–69	184	75.2 ± 22.2	640	83.3 ± 19.3	< 0.001	−8,1	108 (59%)
70+	75	68.3 ± 25.3	1004	85.4 ± 18.0	< 0.001	**−17**,**0**	34 (45%)
Age group	Number of patients	KOOS ADL*	Number of peers	KOOS ADL*	P-value	MCID 10 **	Number of recovered patients ***
18–29	70	84.1 ± 18.2	152	91.3 ± 14.1	0.004	−7,2	47 (67%)
30–39	76	79.1 ± 22.4	217	93.0 ± 12.5	< 0.001	**−13**,**9**	45 (59%)
40–49	113	75.5 ± 20.4	350	90.4 ± 15.3	< 0.001	**−14**,**9**	50 (44%)
50–59	236	75.4 ± 22.3	473	85.9 ± 18.4	< 0.001	**−10**,**5**	128 (54%)
60–69	184	78.0 ± 20.1	639	84.8 ± 18.1	< 0.001	−6,8	105 (57%)
70+	75	70.2 ± 22.6	998	84.7 ± 18.1	< 0.001	**−14**,**5**	32 (43%)
Age group	Number of patients	KOOS Sport*	Number of peers	KOOS Sport*	P-value	MCID 9 **	Number of recovered patients ***
18–29	69	50.6 ± 31.9	152	77.8 ± 27.1	< 0.001	**−27**,**2**	24 (35%)
30–39	76	41.8 ± 34.8	217	81.2 ± 25.1	< 0.001	**−39**,**4**	21 (28%)
40–49	111	41.7 ± 31.4	348	77.7 ± 27.3	0.000	**−36**,**0**	29 (26%)
50–59	231	42.1 ± 33.8	469	71.1 ± 29.9	0.000	**−29**,**0**	70 (30%)
60–69	182	43.0 ± 33.3	631	66.5 ± 31.4	0.000	**−23**,**5**	60 (33%)
70+	74	37.6 ± 34.1	982	67.8 ± 31.2	< 0.001	**−30**,**2**	21 (28%)
Age group	Number of patients	KOOS Quality of life*	Number of peers	KOOS Quality of life*	P-value	MCID 14 **	Number of recovered patients ***
18–29	70	59.4 ± 26.7	152	76.9 ± 25.0	< 0.001	**−17**,**5**	33 (47%)
30–39	76	51.7 ± 24.7	217	80.8 ± 23.2	0.000	**−29**,**1**	25 (33%)
40–49	112	50.7 ± 26.0	351	78.5 ± 23.0	0.000	**−27**,**8**	31 (28%)
50–59	236	53.2 ± 29.4	473	73.8 ± 25.9	0.000	**−20**,**6**	99 (42%)
60–69	184	57.7 ± 28.8	640	71.2 ± 26.4	< 0.001	−13,5	85 (46%)
70+	75	49.3 ± 29.2	996	74.6 ± 25.6	< 0.001	**−25**,**3**	27 (36%)

* The values are given as mean and standard deviation.

** The cursive numbers indicate KOOS-scores exceeding the minimalclinically important difference (MCID)

*** The number and percentage of patients that recovered within the MCID range of the outcome scores from age-related peers.

## Discussion

To adequately counsel patients regarding outcome, it is important to understand to what extent they are expected to recover during follow-up. However, it is still unclear how patient-reported outcomes after tibial plateau fractures compare with functional outcome scores of age-related peers from the general population. This multicenter study, which reports patient-reported outcomes from 1101 individuals after tibial plateau fractures, provides a guideline for managing patient expectations by comparing recovery outcomes of operatively and nonoperatively treated patients with age-matched peers from the general population. On average, patients with tibial plateau fractures reported approximately 5% lower patient-reported outcomes for general functioning, pain management and activities of daily living after nonoperative treatment and 10% lower after operative treatment compared to their peers. The most affected domains were sports performance and recreation as well as quality of life, with a decline of 22% after nonoperative treatment and 36% after operative treatment relative to the general population. This effect is particularly pronounced in younger age groups, as they typically start from higher athletic performance levels and experience a substantial reduction in sports capacity.

### Level of recovery as compared to age-related peers

Our study reveals that patients recovering from tibial plateau fractures tend to score slightly lower in general physical functioning, pain management, and activities of daily living compared to age-related peers. The most pronounced impairments in all age groups were in sports and recreation as well as quality of life. Importantly, we present age-stratified insights into functional outcomes, enhancing the clinical relevance of our findings. To our knowledge, no other studies have compared a large multicenter cohort of patients with tibial plateau fractures to age-related peers from the general population. This makes it challenging to directly compare our findings with those of other studies. While some studies report on functional outcomes following tibial plateau fractures, their findings are hard to interpret due to the lack of comparisons with pre-injury functional levels or a surrogate such as age-related peers from the general population [8, 10]. Due to differences in activity level, ambulatory status, pre-existing comorbidities and coping mechanisms in different age groups, it is likely that age is associated with outcome. Literature regarding reference values of the general population supports that age influences the outcome and showed different functional outcome scores between age groups, especially for the domains sports and recreation as well as quality of life [14, 15]. Van den Berg et al., reported on functional outcome scores of 118 operatively and 42 nonoperatively treated patients and compared these with reference data [10]. Their study showed especially for sport lower outcome scores compared to the general population in both treatment groups, but the impact was more pronounced in operatively treated patients. Our study builds on these preliminary findings and presents outcomes from 1101 patients, both conservatively and operatively treated, stratified by age. Our study showed that the actual impact of these fractures is high for all KOOS-subscales in operatively treated patients, especially for sports and recreation as well as quality of life. In addition, our study showed that the impact is high for sport and recreation as well as quality of life in conservatively treated patients. In 2017, Robertson et al. performed a systematic review on return to sport following tibial plateau fractures [21]. They report on whether patients return to sports or not in a wide range of small studies conducted between 1988 and 2016. They found that 70% of patients with tibial plateau fractures returned to some form of sports, but they didn’t report on actual patient-reported outcome scores regarding sports. Our study contributes to this review by including a multicenter cohort larger than the combined total of the 27 studies presented over a period of 28 year in that review. Moreover, we report PROMs and compared them with age-related peers from the general population, providing age- and treatment-stratified analyses of patient-perceived recovery of level of sports. Furthermore, we provided a sub-analysis where we also stratified for the Schatzker classification to show differences in outcome between simple and more complex fractures [16]. Overall, most patients recovering from tibial plateau fractures regain sufficient function to perform well in daily activities. However, many do not return to a level of sports and recreation as well as quality of life comparable to their peers. Nonoperative treated patients, often with minimally displaced fractures, tend to have better recovery outcomes compared to those undergoing operative treatment for more complex fractures. Our study further explores and presents the extent of these recovery outcomes, providing a comprehensive comparison with peers from the general population. Moreover, as the Schatzker classification increases, often indicating more extensive injury, expected recovery outcomes tend to diminish (supplementary data). This study serves as a guideline for managing recovery expectations after tibial plateau fractures across different age groups and fracture types and may facilitate shared decision-making by informing patients and clinicians about expected outcomes.

### Limitations

This study has some strengths and limitations which need to be addressed. First, we acknowledge this study has some selection bias which is inherent to the cross-sectional study design due to nonresponse [22]. We tried to reduce the risk of nonresponse bias by approaching all patients multiple times. The response rate was 55% which is in line with literature regarding postal survey studies [23]. Furthermore, the nonresponse analysis showed only small differences in age, sex and treatment between responders and non-responders and no differences in Schatzker classification, suggesting little impact on the generatability of our findings. Secondly, mean follow-up was 6 ± 4 years and therefore our findings are applicable for mid-term follow-up, whereas impact of these fractures at long-term follow-up (i.e. >20 years) remains unknown. Third, we did not account for fracture severity, sex, or other patient characteristics in our primary analysis, as stratifying by age, sex, treatment, and Schatzker classification would have resulted in groups too small for meaningful comparison—even with a cohort of 1101 patients. To address this, we conducted a sub-analysis stratified by age and Schatzker classification to explore differences in functional outcomes between patients with simple and more complex fracture patterns relative to age-related peers. Lastly, due to the retrospective nature of this study, pre-injury scores were not available. Therefore, we compared the patient-reported functional outcomes with the outcome scores from the general Danish population [15]. Although the Danish population is culturally and demographically quite similar to the Dutch population described in our study, small differences may still be expected. Moreover, for patients who underwent conversion to TKA, we used reference values from patients just before conversion to TKA, to better reflect their functional status at that time. Both these reference values may not fully capture patient-specific recovery but rather reflect recovery at a population level. Therefore, patient-specific recovery should be further investigated in a prospective follow-up study.

## Conclusion

In conclusion, this large multicenter study showed that patients with tibial plateau fractures consistently have lower functional outcome scores compared to age-related peers from the general population, with the differences being most pronounced in younger age groups. Patients treated nonoperatively, often with minimally displaced fractures, generally experience better recovery outcomes than those who undergo surgery for more complex fractures. While most patients regain sufficient function to perform daily activities, tibial plateau fractures significantly impact patient’s ability to perform sports and recreation and diminish quality of life in all age groups. Most patients will not return to their previous level in these domains compared to age-related peers. These findings can help as a guideline to manage patients’ expectations regarding the expected functional outcome during counselling after tibial plateau fractures. Future large prospective multicenter studies are needed, including the patient pre-injury scores, to comprehend more insight into the course of recovery.

## Supplementary Information

Below is the link to the electronic supplementary material.

ESM 1 (DOCX 57.6 KB)

## Data Availability

No datasets were generated or analysed during the current study.
