# Supplementary material for: Most patients with tibial plateau fractures regain function for daily activities but do not return to their pre-injury level of sport: comparison of multicenter cohort of 1101 patients and age-related peers
Source: Eur J Trauma Emerg Surg. 2025 Sep 3;51(1):285. doi: 10.1007/s00068-025-02958-9 (PMC12408669; doi:10.1007/s00068-025-02958-9)
Supplement: Supplementary file 1 — (DOCX 57.6 KB) [file 68_2025_2958_MOESM1_ESM.docx]

**Supplementary data**

**Table 1 The average KOOS-score of each age group stratified by Schatzker classification compared with age-related peers.**

| **Age 18-29** | Outcome score after  Tibial plateau fracture | | Outcome score general population | |  | Difference on a scale of 0-100 | |
| --- | --- | --- | --- | --- | --- | --- | --- |
| Classification | Number of patients | KOOS Symptoms | Number of peers | KOOS Symptoms | P-value* | MCID 9 ** | Number of recovered patients *** |
| Schatzker I | 14 | 77.6 ± 20.9 | 153 | 82.0 ± 18.1 | 0.391 | -4.4 | 10 (71) |
| Schatzker II | 36 | 72.6 ± 18.5 | 153 | 82.0 ± 18.1 | 0.006 | **-9.4** | 18 (50) |
| Schatzker III | 24 | 81.9 ± 15.7 | 153 | 82.0 ± 18.1 | 0.980 | -0.1 | 18 (75) |
| Schatzker IV | 12 | 60.4 ± 20.7 | 153 | 82.0 ± 18.1 | <0.001 | **-21.6** | 3 (25) |
| Schatzker V | 13 | 67.6 ± 28.1 | 153 | 82.0 ± 18.1 | 0.093 | **-14.4** | 7 (54) |
| Schatzker VI | 6 | 63.1 ± 20.3 | 153 | 82.0 ± 18.1 | 0.013 | **-18.9** | 2 (33) |
| Classification | Number of patients | KOOS  Pain | Number of peers | KOOS  Pain | P-value* | MCID 12 ** | Number of recovered patients *** |
| Schatzker I | 14 | 84.6 ± 17.3 | 153 | 85.6 ± 18.2 | 0.844 | -1.0 | 11 (79) |
| Schatzker II | 36 | 76.4 ± 20.4 | 153 | 85.6 ± 18.2 | 0.008 | -9.2 | 22 (61) |
| Schatzker III | 24 | 80.7 ± 15.9 | 153 | 85.6 ± 18.2 | 0.214 | -4.9 | 16 (67) |
| Schatzker IV | 12 | 64.6 ± 25.0 | 153 | 85.6 ± 18.2 | 0.015 | **-21** | 5 (42) |
| Schatzker V | 13 | 76.2 ± 22.8 | 153 | 85.6 ± 18.2 | 0.082 | -9.4 | 8 (62) |
| Schatzker VI | 6 | 72.0 ± 14.5 | 153 | 85.6 ± 18.2 | 0.073 | **-13.6** | 2 (33) |
| Classification | Number of patients | KOOS  ADL | Number of peers | KOOS  ADL | P-value* | MCID 10 ** | Number of recovered patients *** |
| Schatzker I | 14 | 91.3 ± 18.5 | 152 | 91.3 ± 14.1 | 1.000 | 0.0 | 13 (93) |
| Schatzker II | 36 | 82.8 ± 18.3 | 152 | 91.3 ± 14.1 | 0.012 | -8.5 | 22 (61) |
| Schatzker III | 24 | 86.3 ± 16.1 | 152 | 91.3 ± 14.1 | 0.115 | -5.0 | 17 (71) |
| Schatzker IV | 12 | 75.4 ± 22.4 | 152 | 91.3 ± 14.1 | <0.001 | **-15.9** | 6 (50) |
| Schatzker V | 13 | 83.2 ± 21.7 | 152 | 91.3 ± 14.1 | 0.209 | -8.1 | 9 (69) |
| Schatzker VI | 6 | 84.9 ± 10.4 | 152 | 91.3 ± 14.1 | 0.274 | -6.4 | 4 (67) |
| Classification | Number of patients | KOOS  Sport/Rec | Number of peers | KOOS  Sport/Rec | P-value* | MCID 9 ** | Number of recovered patients *** |
| Schatzker I | 14 | 70.2 ± 28.3 | 152 | 77.8 ± 27.1 | 0.319 | -7.6 | 7 (50) |
| Schatzker II | 36 | 49.1 ± 32.9 | 152 | 77.8 ± 27.1 | <0.001 | **-28.7** | 12 (33) |
| Schatzker III | 24 | 56.9 ± 31.1 | 152 | 77.8 ± 27.1 | <0.001 | **-20.9** | 11 (46) |
| Schatzker IV | 12 | 41.7 ± 32.7 | 152 | 77.8 ± 27.1 | <0.001 | **-36.1** | 4 (33) |
| Schatzker V | 13 | 53.8 ± 36.3 | 152 | 77.8 ± 27.1 | 0.036 | **-24.0** | 6 (46) |
| Schatzker VI | 5 | 47.0 ± 30.3 | 152 | 77.8 ± 27.1 | 0.014 | **-30.8** | 1 (20) |
| Classification | Number of patients | KOOS  Quality of life | Number of peers | KOOS  Quality of life | P-value* | MCID 14 ** | Number of recovered patients *** |
| Schatzker I | 14 | 70.1 ± 18.2 | 152 | 76.9 ± 25.0 | 0.322 | -6.8 | 9 (64) |
| Schatzker II | 36 | 56.8 ± 26.3 | 152 | 76.9 ± 25.0 | <0.001 | **-20.1** | 14 (39) |
| Schatzker III | 24 | 70.8 ± 21.2 | 152 | 76.9 ± 25.0 | 0.259 | -6.1 | 15 (63) |
| Schatzker IV | 12 | 48.3 ± 30.9 | 152 | 76.9 ± 25.0 | <0.001 | **-28.6** | 4 (33) |
| Schatzker V | 13 | 64.9 ± 28.7 | 152 | 76.9 ± 25.0 | 0.103 | **-12.0** | 8 (62) |
| Schatzker VI | 6 | 46.9 ± 27.3 | 152 | 76.9 ± 25.0 | 0.005 | **-30.0** | 1 (17) |

** The values are given as mean and standard deviation.*

*** The cursive numbers indicate KOOS-scores exceeding the minimal clinically important difference (MCID)*

**** The number and percentage of patients that recovered within the MCID range of the outcome scores from age-related peers.*

| **Age 30-39** | Outcome score after  Tibial plateau fracture | | Outcome score general population | |  | Difference on a scale of 0-100 | |
| --- | --- | --- | --- | --- | --- | --- | --- |
| Classification | Number of patients | KOOS Symptoms | Number of peers | KOOS Symptoms | P-value* | MCID 9 ** | Number of recovered patients *** |
| Schatzker I | 10 | 88.6 ± 13.1 | 218 | 86.3 ± 14.7 | 0.628 | +2.3 | 8 (80) |
| Schatzker II | 26 | 69.0 ± 19.9 | 218 | 86.3 ± 14.7 | <0.001 | **-17.3** | 9 (35) |
| Schatzker III | 28 | 79.8 ± 21.1 | 218 | 86.3 ± 14.7 | 0.124 | -6.5 | 18 (64) |
| Schatzker IV | 24 | 72.5 ± 20.2 | 218 | 86.3 ± 14.7 | 0.003 | **-13.8** | 10 (42) |
| Schatzker V | 4 | 71.4 ± 27.8 | 218 | 86.3 ± 14.7 | 0.363 | **-14.9** | 2 (50) |
| Schatzker VI | 10 | 69.8 ± 25.3 | 218 | 86.3 ± 14.7 | 0.070 | **-16.5** | 4 (40) |
| Classification | Number of patients | KOOS  Pain | Number of peers | KOOS  Pain | P-value* | MCID 12 ** | Number of recovered patients *** |
| Schatzker I | 10 | 85.0 ± 14.4 | 218 | 89.4 ± 15.3 | 0.374 | -4.4 | 8 (80) |
| Schatzker II | 26 | 75.5 ± 22.3 | 218 | 89.4 ± 15.3 | 0.004 | **-13.9** | 15 (58) |
| Schatzker III | 28 | 81.7 ± 19.7 | 218 | 89.4 ± 15.3 | 0.055 | -7.7 | 20 (71) |
| Schatzker IV | 24 | 72.3 ± 23.9 | 218 | 89.4 ± 15.3 | 0.002 | **-17.1** | 10 (42) |
| Schatzker V | 4 | 68.8 ± 25.2 | 218 | 89.4 ± 15.3 | 0.201 | **-20.6** | 2 (50) |
| Schatzker VI | 10 | 70.9 ± 29.7 | 218 | 89.4 ± 15.3 | 0.081 | **-18.5** | 6 (60) |
| Classification | Number of patients | KOOS  ADL | Number of peers | KOOS  ADL | P-value* | MCID 10 ** | Number of recovered patients *** |
| Schatzker I | 10 | 88.5 ± 15.4 | 217 | 93.0 ± 12.5 | 0.272 | -4.5 | 8 (80) |
| Schatzker II | 26 | 78.8 ± 21.1 | 217 | 93.0 ± 12.5 | 0.002 | **-14.2** | 14 (54) |
| Schatzker III | 28 | 86.6 ± 18.0 | 217 | 93.0 ± 12.5 | 0.078 | -6.4 | 22 (79) |
| Schatzker IV | 24 | 78.3 ± 23.1 | 217 | 93.0 ± 12.5 | 0.005 | **-14.7** | 13 (54) |
| Schatzker V | 4 | 71.3 ± 32.7 | 217 | 93.0 ± 12.5 | 0.276 | **-21.7** | 2 (50) |
| Schatzker VI | 10 | 73.6 ± 27.8 | 217 | 93.0 ± 12.5 | 0.055 | **-19.4** | 4 (40) |
| Classification | Number of patients | KOOS  Sport/Rec | Number of peers | KOOS  Sport/Rec | P-value* | MCID 9 ** | Number of recovered patients *** |
| Schatzker I | 10 | 55.0 ± 38.2 | 217 | 81.2 ± 25.1 | 0.059 | **-26.2** | 5 (50) |
| Schatzker II | 26 | 33.8 ± 34.0 | 217 | 81.2 ± 25.1 | <0.001 | **-47.4** | 5 (19) |
| Schatzker III | 27 | 56.1 ± 35.4 | 217 | 81.2 ± 25.1 | 0.001 | **-25.1** | 11 (41) |
| Schatzker IV | 24 | 47.0 ± 36.9 | 217 | 81.2 ± 25.1 | <0.001 | **-34.2** | 8 (33) |
| Schatzker V | 4 | 53.4 ± 41.7 | 217 | 81.2 ± 25.1 | 0.275 | **-27.8** | 2 (50) |
| Schatzker VI | 10 | 36.1 ± 36.7 | 217 | 81.2 ± 25.1 | 0.004 | **-45.1** | 2 (20) |
| Classification | Number of patients | KOOS  Quality of life | Number of peers | KOOS  Quality of life | P-value* | MCID 14 ** | Number of recovered patients *** |
| Schatzker I | 10 | 58.1 ± 26.7 | 217 | 80.8 ± 23.2 | 0.003 | **-22.7** | 5 (50) |
| Schatzker II | 26 | 50.2 ± 20.5 | 217 | 80.8 ± 23.2 | <0.001 | **-30.6** | 6 (23) |
| Schatzker III | 27 | 61.0 ± 25.2 | 217 | 80.8 ± 23.2 | <0.001 | **-19.8** | 12 (44) |
| Schatzker IV | 24 | 54.4 ± 27.5 | 217 | 80.8 ± 23.2 | <0.001 | **-26.3** | 10 (42) |
| Schatzker V | 4 | 56.3 ± 22.2 | 217 | 80.8 ± 23.2 | 0.037 | **-24.5** | 2 (50) |
| Schatzker VI | 10 | 44.0 ± 28.7 | 217 | 80.8 ± 23.2 | <0.001 | **-36.8** | 3 (30) |

** The values are given as mean and standard deviation.*

*** The cursive numbers indicate KOOS-scores exceeding the minimal clinically important difference (MCID)*

**** The number and percentage of patients that recovered within the MCID range of the outcome scores from age-related peers.*

| **Age 40-49** | Outcome score after  Tibial plateau fracture | | Outcome score general population | |  | Difference on a scale of 0-100 | |
| --- | --- | --- | --- | --- | --- | --- | --- |
| Classification | Number of patients | KOOS Symptoms | Number of peers | KOOS Symptoms | P-value* | MCID 9 ** | Number of recovered patients *** |
| Schatzker I | 13 | 81.6 ± 15.3 | 351 | 85.5 ± 16.3 | 0.397 | -3.9 | 8 (62) |
| Schatzker II | 58 | 71.3 ± 20.6 | 351 | 85.5 ± 16.3 | <0.001 | **-14.2** | 28 (48) |
| Schatzker III | 26 | 82.0 ± 17.3 | 351 | 85.5 ± 16.3 | 0.293 | -3.5 | 18 (69) |
| Schatzker IV | 29 | 70.6 ± 19.5 | 351 | 85.5 ± 16.3 | <0.001 | **-14.9** | 11 (38) |
| Schatzker V | 10 | 73.4 ± 21.9 | 351 | 85.5 ± 16.3 | 0.023 | **-12.1** | 5 (50) |
| Schatzker VI | 22 | 67.2 ± 26.7 | 351 | 85.5 ± 16.3 | 0.004 | **-18.3** | 10 (45) |
| Classification | Number of patients | KOOS  Pain | Number of peers | KOOS  Pain | P-value* | MCID 12 ** | Number of recovered patients *** |
| Schatzker I | 13 | 80.0 ± 19.9 | 351 | 87.2 ± 17.1 | 0.139 | -7.2 | 7 (54) |
| Schatzker II | 58 | 74.0 ± 21.7 | 351 | 87.2 ± 17.1 | <0.001 | **-13.2** | 30 (52) |
| Schatzker III | 26 | 84.3 ± 19.2 | 351 | 87.2 ± 17.1 | 0.409 | -2.9 | 19 (73) |
| Schatzker IV | 29 | 71.8 ± 19.8 | 351 | 87.2 ± 17.1 | <0.001 | **-15.4** | 12 (41) |
| Schatzker V | 10 | 77.8 ± 22.5 | 351 | 87.2 ± 17.1 | 0.090 | -9.4 | 6 (60) |
| Schatzker VI | 22 | 67.0 ± 26.5 | 351 | 87.2 ± 17.1 | 0.002 | **-20.2** | 9 (41) |
| Classification | Number of patients | KOOS  ADL | Number of peers | KOOS  ADL | P-value* | MCID 10 ** | Number of recovered patients *** |
| Schatzker I | 13 | 83.8 ± 19.0 | 350 | 90.4 ± 15.3 | 0.131 | -6.6 | 9 (69) |
| Schatzker II | 58 | 77.1 ± 20.1 | 350 | 90.4 ± 15.3 | <0.001 | **-13.3** | 29 (50) |
| Schatzker III | 26 | 86.6 ± 16.0 | 350 | 90.4 ± 15.3 | 0.224 | -3.8 | 17 (65) |
| Schatzker IV | 29 | 75.3 ± 19.2 | 350 | 90.4 ± 15.3 | <0.001 | **-15.1** | 11 (38) |
| Schatzker V | 10 | 80.2 ± 21.2 | 350 | 90.4 ± 15.3 | 0.164 | **-10.2** | 6 (60) |
| Schatzker VI | 22 | 72.1 ± 22.5 | 350 | 90.4 ± 15.3 | 0.001 | **-18.3** | 8 (36) |
| Classification | Number of patients | KOOS  Sport/Rec | Number of peers | KOOS  Sport/Rec | P-value* | MCID 9 ** | Number of recovered patients *** |
| Schatzker I | 13 | 58.8 ± 31.7 | 348 | 77.7 ± 27.3 | 0.015 | **-18.9** | 5 (38) |
| Schatzker II | 56 | 40.0 ± 28.8 | 348 | 77.7 ± 27.3 | 0.000 | **-37.7** | 11 (20) |
| Schatzker III | 26 | 61.8 ± 35.6 | 348 | 77.7 ± 27.3 | 0.034 | **-15.9** | 14 (54) |
| Schatzker IV | 29 | 43.5 ± 29.9 | 348 | 77.7 ± 27.3 | <0.001 | **-34.2** | 9 (31) |
| Schatzker V | 10 | 55.6 ± 42.6 | 348 | 77.7 ± 27.3 | 0.137 | **-22.1** | 5 (50) |
| Schatzker VI | 22 | 33.8 ± 31.9 | 348 | 77.7 ± 27.3 | <0.001 | **-43.9** | 4 (18) |
| Classification | Number of patients | KOOS  Quality of life | Number of peers | KOOS  Quality of life | P-value* | MCID 14 ** | Number of recovered patients *** |
| Schatzker I | 13 | 61.1 ± 26.5 | 351 | 78.5 ± 23.0 | 0.008 | **-17.4** | 5 (38) |
| Schatzker II | 57 | 50.1 ± 25.4 | 351 | 78.5 ± 23.0 | <0.001 | **-28.4** | 15 (26) |
| Schatzker III | 26 | 68.1 ± 25.0 | 351 | 78.5 ± 23.0 | 0.028 | -10.4 | 15 (58) |
| Schatzker IV | 29 | 49.9 ± 26.3 | 351 | 78.5 ± 23.0 | <0.001 | **-28.6** | 8 (28) |
| Schatzker V | 10 | 64.0 ± 31.6 | 351 | 78.5 ± 23.0 | 0.183 | **-14.5** | 5 (50) |
| Schatzker VI | 22 | 46.3 ± 26.3 | 351 | 78.5 ± 23.0 | <0.001 | **-32.2** | 5 (23) |

** The values are given as mean and standard deviation.*

*** The cursive numbers indicate KOOS-scores exceeding the minimal clinically important difference (MCID)*

**** The number and percentage of patients that recovered within the MCID range of the outcome scores from age-related peers.*

| **Age 50-59** | Outcome score after  Tibial plateau fracture | | Outcome score general population | |  | Difference on a scale of 0-100 | |
| --- | --- | --- | --- | --- | --- | --- | --- |
| Classification | Number of patients | KOOS Symptoms | Number of peers | KOOS Symptoms | P-value* | MCID 9 ** | Number of recovered patients *** |
| Schatzker I | 21 | 77.7 ± 17.6 | 473 | 83.7 ± 17.3 | 0.121 | -6.0 | 14 (67) |
| Schatzker II | 114 | 79.1 ± 20.0 | 473 | 83.7 ± 17.3 | 0.025 | -4.6 | 73 (64) |
| Schatzker III | 72 | 78.8 ± 20.8 | 473 | 83.7 ± 17.3 | 0.061 | -4.9 | 47 (65) |
| Schatzker IV | 37 | 71.9 ± 22.2 | 473 | 83.7 ± 17.3 | 0.003 | **-11.8** | 18 (49) |
| Schatzker V | 28 | 69.3 ± 22.1 | 473 | 83.7 ± 17.3 | 0.002 | **-14.4** | 11 (39) |
| Schatzker VI | 57 | 64.7 ± 21.5 | 473 | 83.7 ± 17.3 | <0.001 | **-19.0** | 18 (32) |
| Classification | Number of patients | KOOS  Pain | Number of peers | KOOS  Pain | P-value* | MCID 12 ** | Number of recovered patients *** |
| Schatzker I | 21 | 79.8 ± 18.7 | 473 | 84.2 ± 19.0 | 0.299 | -4.4 | 15 (71) |
| Schatzker II | 114 | 79.3 ± 21.6 | 473 | 84.2 ± 19.0 | 0.028 | -4.9 | 76 (67) |
| Schatzker III | 72 | 77.5 ± 24.4 | 473 | 84.2 ± 19.0 | 0.028 | -6.7 | 44 (61) |
| Schatzker IV | 37 | 75.7 ± 24.4 | 473 | 84.2 ± 19.0 | 0.045 | -8.5 | 24 (65) |
| Schatzker V | 28 | 69.5 ± 25.1 | 473 | 84.2 ± 19.0 | 0.005 | **-14.7** | 15 (54) |
| Schatzker VI | 57 | 65.2 ± 24.5 | 473 | 84.2 ± 19.0 | <0.001 | **-19.0** | 23 (40) |
| Classification | Number of patients | KOOS  ADL | Number of peers | KOOS  ADL | P-value* | MCID 10 ** | Number of recovered patients *** |
| Schatzker I | 21 | 80.4 ± 16.6 | 473 | 85.9 ± 18.4 | 0.179 | -5.5 | 15 (71) |
| Schatzker II | 114 | 80.4 ± 21.0 | 473 | 85.9 ± 18.4 | 0.011 | -5.5 | 70 (61) |
| Schatzker III | 72 | 80.5 ± 22.4 | 473 | 85.9 ± 18.4 | 0.055 | -5.4 | 47 (65) |
| Schatzker IV | 37 | 75.0 ± 24.6 | 473 | 85.9 ± 18.4 | 0.012 | **-10.9** | 24 (65) |
| Schatzker V | 28 | 74.4 ± 20.5 | 473 | 85.9 ± 18.4 | 0.001 | **-11.5** | 15 (54) |
| Schatzker VI | 57 | 67.5 ± 22.2 | 473 | 85.9 ± 18.4 | <0.001 | **-18.4** | 21 (37) |
| Classification | Number of patients | KOOS  Sport/Rec | Number of peers | KOOS  Sport/Rec | P-value* | MCID 9 ** | Number of recovered patients *** |
| Schatzker I | 21 | 44.2 ± 32.9 | 469 | 71.1 ± 29.9 | <0.001 | **-26.9** | 6 (29) |
| Schatzker II | 111 | 50.8 ± 35.0 | 469 | 71.1 ± 29.9 | <0.001 | **-20.3** | 45 (41) |
| Schatzker III | 72 | 53.4 ± 35.4 | 469 | 71.1 ± 29.9 | <0.001 | **-17.7** | 32 (44) |
| Schatzker IV | 36 | 42.0 ± 34.5 | 469 | 71.1 ± 29.9 | <0.001 | **-29.1** | 10 (28) |
| Schatzker V | 27 | 33.3 ± 33.8 | 469 | 71.1 ± 29.9 | <0.001 | **-37.8** | 6 (22) |
| Schatzker VI | 55 | 30.3 ± 28.3 | 469 | 71.1 ± 29.9 | 0.000 | **-40.8** | 8 (15) |
| Classification | Number of patients | KOOS  Quality of life | Number of peers | KOOS  Quality of life | P-value* | MCID 14 ** | Number of recovered patients *** |
| Schatzker I | 21 | 58.4 ± 23.9 | 473 | 73.8 ± 25.9 | 0.008 | **-15.4** | 9 (43) |
| Schatzker II | 114 | 61.8 ± 29.5 | 473 | 73.8 ± 25.9 | <0.001 | -12.0 | 66 (58) |
| Schatzker III | 72 | 63.1 ± 29.6 | 473 | 73.8 ± 25.9 | 0.001 | -10.7 | 40 (56) |
| Schatzker IV | 37 | 50.3 ± 27.9 | 473 | 73.8 ± 25.9 | <0.001 | **-23.5** | 12 (32) |
| Schatzker V | 28 | 50.1 ± 30.4 | 473 | 73.8 ± 25.9 | <0.001 | **-23.7** | 10 (36) |
| Schatzker VI | 57 | 43.9 ± 26.8 | 473 | 73.8 ± 25.9 | <0.001 | **-29.9** | 16 (28) |

** The values are given as mean and standard deviation.*

*** The cursive numbers indicate KOOS-scores exceeding the minimal clinically important difference (MCID)*

**** The number and percentage of patients that recovered within the MCID range of the outcome scores from age-related peers.*

| **Age 60-69** | Outcome score after  Tibial plateau fracture | | Outcome score general population | |  | Difference on a scale of 0-100 | |
| --- | --- | --- | --- | --- | --- | --- | --- |
| Classification | Number of patients | KOOS Symptoms | Number of peers | KOOS Symptoms | P-value* | MCID 9 ** | Number of recovered patients *** |
| Schatzker I | 11 | 84.9 ± 18.7 | 640 | 83.6 ± 17.3 | 0.805 | +1.3 | 8 (73) |
| Schatzker II | 105 | 81.9 ± 19.6 | 640 | 83.6 ± 17.3 | 0.404 | -1.7 | 76 (72) |
| Schatzker III | 65 | 77.9 ± 19.3 | 640 | 83.6 ± 17.3 | 0.013 | -5.7 | 41 (63) |
| Schatzker IV | 36 | 73.8 ± 20.4 | 640 | 83.6 ± 17.3 | 0.001 | -9.8 | 17 (47) |
| Schatzker V | 12 | 80.4 ± 20.5 | 640 | 83.6 ± 17.3 | 0.527 | -3.2 | 7 (58) |
| Schatzker VI | 48 | 70.3 ± 19.5 | 640 | 83.6 ± 17.3 | <0.001 | **-13.3** | 22 (46) |
| Classification | Number of patients | KOOS  Pain | Number of peers | KOOS  Pain | P-value* | MCID 12 ** | Number of recovered patients *** |
| Schatzker I | 11 | 82.6 ± 21.4 | 640 | 83.3 ± 19.3 | 0.905 | -0.7 | 8 (73) |
| Schatzker II | 105 | 81.8 ± 20.1 | 640 | 83.3 ± 19.3 | 0.463 | -1.5 | 73 (70) |
| Schatzker III | 65 | 77.8 ± 22.0 | 640 | 83.3 ± 19.3 | 0.031 | -5.5 | 42 (65) |
| Schatzker IV | 35 | 71.7 ± 24.1 | 640 | 83.3 ± 19.3 | 0.008 | -11.6 | 19 (54) |
| Schatzker V | 12 | 76.7 ± 23.6 | 640 | 83.3 ± 19.3 | 0.243 | -6.6 | 8 (67) |
| Schatzker VI | 48 | 70.0 ± 23.7 | 640 | 83.3 ± 19.3 | <0.001 | **-13.3** | 23 (48) |
| Classification | Number of patients | KOOS  ADL | Number of peers | KOOS  ADL | P-value* | MCID 10 ** | Number of recovered patients *** |
| Schatzker I | 11 | 82.8 ± 21.8 | 639 | 84.8 ± 18.1 | 0.717 | -2.0 | 7 (64) |
| Schatzker II | 105 | 84.1 ± 18.0 | 639 | 84.8 ± 18.1 | 0.713 | -0.7 | 72 (69) |
| Schatzker III | 65 | 79.3 ± 20.4 | 639 | 84.8 ± 18.1 | 0.021 | -5.5 | 39 (60) |
| Schatzker IV | 36 | 74.0 ± 21.6 | 639 | 84.8 ± 18.1 | <0.001 | **-10.8** | 18 (50) |
| Schatzker V | 12 | 83.7 ± 18.4 | 639 | 84.8 ± 18.1 | 0.835 | -1.1 | 9 (75) |
| Schatzker VI | 48 | 72.2 ± 21.1 | 639 | 84.8 ± 18.1 | <0.001 | **-12.6** | 23 (48) |
| Classification | Number of patients | KOOS  Sport/Rec | Number of peers | KOOS  Sport/Rec | P-value* | MCID 9 ** | Number of recovered patients *** |
| Schatzker I | 9 | 55.1 ± 34.6 | 631 | 66.5 ± 31.4 | 0.281 | **-11.4** | 4 (44) |
| Schatzker II | 102 | 56.3 ± 33.4 | 631 | 66.5 ± 31.4 | 0.003 | **-10.2** | 53 (52) |
| Schatzker III | 63 | 51.8 ± 34.7 | 631 | 66.5 ± 31.4 | <0.001 | **-14.7** | 29 (46) |
| Schatzker IV | 36 | 40.1 ± 33.5 | 631 | 66.5 ± 31.4 | <0.001 | **-26.4** | 9 (25) |
| Schatzker V | 12 | 51.4 ± 34.2 | 631 | 66.5 ± 31.4 | 0.100 | **-15.1** | 5 (42) |
| Schatzker VI | 48 | 31.7 ± 29.1 | 631 | 66.5 ± 31.4 | <0.001 | **-34.8** | 10 (21) |
| Classification | Number of patients | KOOS  Quality of life | Number of peers | KOOS  Quality of life | P-value* | MCID 14 ** | Number of recovered patients *** |
| Schatzker I | 11 | 67.8 ± 34.2 | 640 | 71.2 ± 26.4 | 0.674 | -3.4 | 7 (64) |
| Schatzker II | 105 | 66.4 ± 28.2 | 640 | 71.2 ± 26.4 | 0.088 | -4.8 | 62 (59) |
| Schatzker III | 65 | 64.9 ± 28.1 | 640 | 71.2 ± 26.4 | 0.069 | -6.3 | 38 (58) |
| Schatzker IV | 36 | 56.1 ± 29.2 | 640 | 71.2 ± 26.4 | <0.001 | **-15.1** | 14 (39) |
| Schatzker V | 12 | 64.0 ± 29.2 | 640 | 71.2 ± 26.4 | 0.351 | -7.2 | 7 (58) |
| Schatzker VI | 48 | 50.5 ± 27.6 | 640 | 71.2 ± 26.4 | <0.001 | **-20.7** | 18 (38) |

** The values are given as mean and standard deviation.*

*** The cursive numbers indicate KOOS-scores exceeding the minimal clinically important difference (MCID)*

**** The number and percentage of patients that recovered within the MCID range of the outcome scores from age-related peers.*

| **Age 70+** | Outcome score after  Tibial plateau fracture | | Outcome score general population | |  | Difference on a scale of 0-100 | |
| --- | --- | --- | --- | --- | --- | --- | --- |
| Classification | Number of patients | KOOS  Symptoms | Number of peers | KOOS  Symptoms | P-value* | MCID 9 ** | Number of recovered patients *** |
| Schatzker I | 8 | 74.8 ± 25.4 | 1007 | 87.0 ± 16.2 | 0.217 | **-12.2** | 5 (63) |
| Schatzker II | 45 | 76.0 ± 24.0 | 1007 | 87.0 ± 16.2 | 0.004 | **-11.0** | 26 (58) |
| Schatzker III | 33 | 83.6 ± 21.9 | 1007 | 87.0 ± 16.2 | 0.383 | -3.4 | 24 (73) |
| Schatzker IV | 20 | 67.0 ± 20.3 | 1007 | 87.0 ± 16.2 | <0.001 | **-20.0** | 7 (35) |
| Schatzker V | 5 | 83.3 ± 20.3 | 1007 | 87.0 ± 16.2 | 0.611 | -3.7 | 3 (60) |
| Schatzker VI | 16 | 74.1 ± 19.8 | 1007 | 87.0 ± 16.2 | 0.002 | **-12.9** | 8 (50) |
| Classification | Number of patients | KOOS  Pain | Number of peers | KOOS  Pain | P-value* | MCID 12 ** | Number of recovered patients *** |
| Schatzker I | 8 | 77.5 ± 22.9 | 1004 | 85.4 ± 18.0 | 0.218 | -7.9 | 4 (50) |
| Schatzker II | 45 | 75.9 ± 25.6 | 1004 | 85.4 ± 18.0 | 0.018 | -9.5 | 28 (62) |
| Schatzker III | 33 | 85.1 ± 20.4 | 1004 | 85.4 ± 18.0 | 0.925 | -0.3 | 26 (79) |
| Schatzker IV | 20 | 60.3 ± 24.6 | 1004 | 85.4 ± 18.0 | <0.001 | **-25.1** | 5 (25) |
| Schatzker V | 5 | 84.6 ± 22.8 | 1004 | 85.4 ± 18.0 | 0.921 | -0.8 | 4 (80) |
| Schatzker VI | 16 | 72.4 ± 24.9 | 1004 | 85.4 ± 18.0 | 0.055 | **-13.0** | 9 (56) |
| Classification | Number of patients | KOOS  ADL | Number of peers | KOOS  ADL | P-value* | MCID 10 ** | Number of recovered patients *** |
| Schatzker I | 8 | 76.5 ± 21.7 | 998 | 84.7 ± 18.1 | 0.203 | -8.2 | 4 (50) |
| Schatzker II | 45 | 76.4 ± 24.3 | 998 | 84.7 ± 18.1 | 0.028 | -8.3 | 27 (60) |
| Schatzker III | 33 | 79.1 ± 28.8 | 998 | 84.7 ± 18.1 | 0.275 | -5.6 | 24 (73) |
| Schatzker IV | 20 | 63.0 ± 22.0 | 998 | 84.7 ± 18.1 | <0.001 | **-21.7** | 5 (25) |
| Schatzker V | 5 | 84.8 ± 18.6 | 998 | 84.7 ± 18.1 | 0.990 | +0.1 | 4 (80) |
| Schatzker VI | 16 | 72.4 ± 21.7 | 998 | 84.7 ± 18.1 | 0.007 | **-12.3** | 7 (44) |
| Classification | Number of patients | KOOS  Sport/Rec | Number of peers | KOOS  Sport/Rec | P-value* | MCID 9 ** | Number of recovered patients *** |
| Schatzker I | 8 | 45.0 ± 40.0 | 982 | 67.8 ± 31.2 | 0.040 | **-22.8** | 3 (38) |
| Schatzker II | 42 | 46.6 ± 37.0 | 982 | 67.8 ± 31.2 | <0.001 | **-21.2** | 18 (43) |
| Schatzker III | 26 | 63.7 ± 36.6 | 982 | 67.8 ± 31.2 | 0.510 | -4.1 | 16 (62) |
| Schatzker IV | 20 | 31.5 ± 32.5 | 982 | 67.8 ± 31.2 | <0.001 | **-36.3** | 4 (20) |
| Schatzker V | 5 | 47.2 ± 34.7 | 982 | 67.8 ± 31.2 | 0.141 | **-20.6** | 2 (40) |
| Schatzker VI | 16 | 39.1 ± 36.3 | 982 | 67.8 ± 31.2 | <0.001 | **-28.7** | 5 (31) |
| Classification | Number of patients | KOOS  Quality of life | Number of peers | KOOS  Quality of life | P-value* | MCID 14 ** | Number of recovered patients *** |
| Schatzker I | 8 | 59.6 ± 31.4 | 996 | 74.6 ± 25.6 | 0.100 | **-15.0** | 4 (50) |
| Schatzker II | 45 | 59.0 ± 33.7 | 996 | 74.6 ± 25.6 | 0.004 | **-15.6** | 23 (51) |
| Schatzker III | 32 | 77.3 ± 25.4 | 996 | 74.6 ± 25.6 | 0.557 | -2.7 | 24 (75) |
| Schatzker IV | 20 | 44.4 ± 28.7 | 996 | 74.6 ± 25.6 | <0.001 | **-30.2** | 5 (25) |
| Schatzker V | 5 | 61.7 ± 27.1 | 996 | 74.6 ± 25.6 | 0.261 | -12.9 | 3 (60) |
| Schatzker VI | 16 | 48.2 ± 30.1 | 996 | 74.6 ± 25.6 | <0.001 | **-26.4** | 6 (38) |

** The values are given as mean and standard deviation.*

*** The cursive numbers indicate KOOS-scores exceeding the minimal clinically important difference (MCID)*

**** The number and percentage of patients that recovered within the MCID range of the outcome scores from age-related peers.*
